# Predictors of impaired growth during the first year of life: findings from a longitudinal cohort study in Pakistan

**DOI:** 10.3389/fnut.2026.1912329

**Published:** 2026-08-17

**Authors:** Ahsan Saidal, Mustajab Ghani, Alaa’ Lutfi Melhem, Maria Ishaq Khattak, Qarib Ullah, Bibi Hajira, Saima Shaheen, Kalsoom Tariq, Ziad Al Nabhani, Simon C. Andrews, Hani A. Alfheeaid, Muhammad Shahzad

**Affiliations:** 1 KMU Institute of Health Sciences, Swat, Pakistan; 2 Faculty of Dentistry, Zarqa University, Zarqa, Jordan; 3 Institute of Public Health and Social Sciences, Khyber Medical University Peshawar, Peshawar, Pakistan; 4 Institute of Basic Medical Sciences, Khyber Medical University, Peshawar, Pakistan; 5 Department of Biochemistry, Khyber Girls Medical College, Peshawar, Pakistan; 6 Department of Visceral Surgery and Medicine, Bern University Hospital, Bern, Switzerland; 7 Maurice Müller Laboratories, Department for Biomedical Research, University of Bern, Bern, Switzerland; 8 School of Biological Sciences, Health and Life Sciences Building, University of Reading, Reading, United Kingdom; 9 Department of Food Science and Human Nutrition, College of Agriculture and Food, Qassim University, Buraydah, Saudi Arabia

**Keywords:** complementary feeding, exclusive breastfeeding, infant growth, malnutrition, maternal education, stunting, underweight

## Abstract

**Background:**

This study aimed to prospectively identify sociodemographic, maternal and child-related predictors of linear growth (height-for-age-z-score, HAZ) and underweight status (weight-for-age- z-score, WAZ) at 12 months among infants from the CHAMP longitudinal cohort in Pakistan.

**Methods:**

We analysed data from the Child Health and Microbiome Development Study (CHAMP), a prospective cohort of 70 mother–infant dyads (72 infants, including one set of triplets) recruited within 0–28 days postpartum from rural District Swat, Pakistan, and followed at 3, 6 and 12 months, using sociodemographic, anthropometric, dietary intake and morbidity data. Weight-for-age (WAZ) and height-for-age (HAZ) z-scores were computed against the 2006 WHO Child Growth Standards. A time-staged multivariable linear regression models were fitted separately for four domains (sociodemographic, maternal, infant morbidity and infant feeding) with WAZ and HAZ at 12 months as primary outcomes. All models were adjusted for infant sex and maternal education.

**Results:**

Anthropometric assessment showed a sharp decline in WAZ between recruitment and 3-months with partial recovery thereafter, whereas HAZ improved initially but declined again at 6 and 12 months. Among all measured predictors, maternal education emerged as the most consistent upstream correlate of growth, showing positive associations with both WAZ and HAZ across sociodemographic, maternal, morbidity and feeding models. In addition, feeding practice at 6 months provided an overall statistical significance (WAZ: *R*^2^ = 0.159, *p* = 0.030; HAZ: *R*^2^ = 0.157, *p* = 0.032) with respect to growth. Within this model, exclusive breastfeeding for less than 6 months was associated with lower HAZ (*B* = −0.613, 95% CI: −1.166 to −0.060, *p* = 0.030), while egg and/or flesh food consumption showed a positive but borderline association with WAZ (*B* = 0.550, 95% CI: −0.106 to 1.205, *p* = 0.099).

**Conclusion:**

Maternal education and infant feeding practices (specifically, not maintaining exclusive breastfeeding to 6 months and the consumption of animal-sourced foods at 6 months) were associated with infant growth during the first year of life. The exclusive breastfeeding association was attenuated after adjustment for baseline height-for-age and should be regarded as suggestive. These findings are hypothesis-generating and require confirmation in adequately powered studies.

## 1 Background

Childhood malnutrition is a substantial public health challenge that disproportionately affects children in low- and middle-income countries (1). Pakistan has one of the highest rates of childhood malnutrition globally. The 2018 National Nutrition Survey in Pakistan reported that 40.2% of children under 5 years were stunted, 17.7% were wasted and 28.9% were underweight (2). Stunting, defined as low height-for-age (HAZ < −2 SD), reflects long-term nutritional deprivation and recurrent infections, whereas underweight (low weight-for-age) reflects a combination of acute and chronic malnutrition (3, 4). These conditions are particularly consequential during the first 1,000 days, from conception to 2 years of life, a critical period recognized as the window of opportunity for optimal physical growth, cognitive development and long-term health (5). Impaired linear growth during this critical period often has serious and irreversible consequences, including poor cognitive performance, reduced educational achievement, increased susceptibility to infectious diseases, lower economic productivity in adulthood and a greater risk of non-communicable diseases (NCDs) later in life (6). Furthermore, it perpetuates the intergenerational cycle of malnutrition particularly in poor resource setting (5).

Impaired growth during early life is a multifactorial process shaped by complex interplay of sociodemographic, dietary intake (both maternal and child), environmental and health related factors (7). Among the sociodemographic determinants, maternal education is considered key underlying determinant of child nutritional status within the UNICEF conceptual framework on maternal and child nutrition (8). Within this framework, maternal education plays a crucial role by improving caregiving capacity, healthcare seeking behaviours, hygiene practices and feeding behaviours of the mothers. These pathways are particularly in low resource settings where maternal education is consistently been identified as a strong predictor of child growth (9–12). Similarly, inadequate maternal nutrition during pregnancy and insufficient nutrient intake during infancy can result in fetal growth restriction and postnatal growth faltering (13). Infant and child related factors such as child feeding practices also significantly contribute to impaired growth and malnutrition (14). Recurrent childhood illnesses particularly diarrhoea and respiratory tract infections, further aggravate undernutrition by reducing appetite, impairing nutrient absorption and increasing metabolic requirements (15). These determinants often coexist and interact synergistically, leading to cumulative growth deficit overtime. Evidence from Pakistan also indicates that children exposed to poor dietary diversity, low maternal literacy, household poverty and repeated infections are at substantially higher risk of stunting and underweight during the first 2 years of life, therefore affecting physical growth, cognitive development and long-term health outcomes (13, 16). Despite the high burden of malnutrition in Pakistan, important gaps remain in the existing evidence base. The available studies are mostly cross sectional in nature (16–18), limiting the ability to establish temporal and causal relationship between early life exposure and growth outcomes. Moreover, these factors have been widely studied individually and relatively few studies have simultaneously assessed the influence of sociodemographic factors, maternal characteristics, infant feeding practices, dietary intake and morbidity on growth trajectories (19). In addition, there is a paucity of locally generated, context-specific data that reflect real-world conditions in diverse Pakistani populations, limiting the generalizability of existing findings and their applicability to programme and policy design.

Given the aforementioned issues, there is a need for longitudinal studies that simultaneously evaluate multiple determinants of child growth during early life. The Child Health And Microbiome Development Study from Pakistan (the CHAMP) is a longitudinal cohort study that followed children for gut microbiome development and associated maternal, infant and demographic factors, during the first 2 years of life (20). The CHAMP cohort profile has previously described the baseline characteristics of the study population, including maternal and household characteristics, infant feeding practices, and the nutritional status of infants at recruitment (21). Building on these findings, the present study aimed to prospectively assess the sociodemographic, maternal and infant-related predictors of child growth during the first year of life. By identifying early-life factors associated with growth trajectories, the study provided evidence to better understand pathways leading to growth faltering and to inform evidence based targeted interventions in a resource limited setting.

## 2 Methods

### 2.1 Study design and participants

This is a secondary analysis of the CHAMP cohort, in which 70 mother–infant dyads (72 infants, including one set of triplets) were recruited from District Swat, Pakistan, within 0–28 days postpartum (May–June 2024) and followed prospectively at 3, 6 and 12 months. The final analytical sample at the 12-month follow-up consisted of 70 mothers and 72 infants. The procedures for measuring length and weight have been reported earlier (16). The primary outcomes were WHO growth-standardised z scores at 12 months: weight-for-age (WAZ) and height/length-for-age (HAZ). Infant weight and recumbent length/standing height (as appropriate at age) were measured using standardised CHAMP procedures previously reported. Z scores were calculated using WHO Child Growth Standards (22). Detailed information about the original CHAMP study design, recruitment strategy and data collection methods have been published elsewhere (20).

### 2.2 Conceptual framework and possible associations with impaired growth

**Figure 1:**
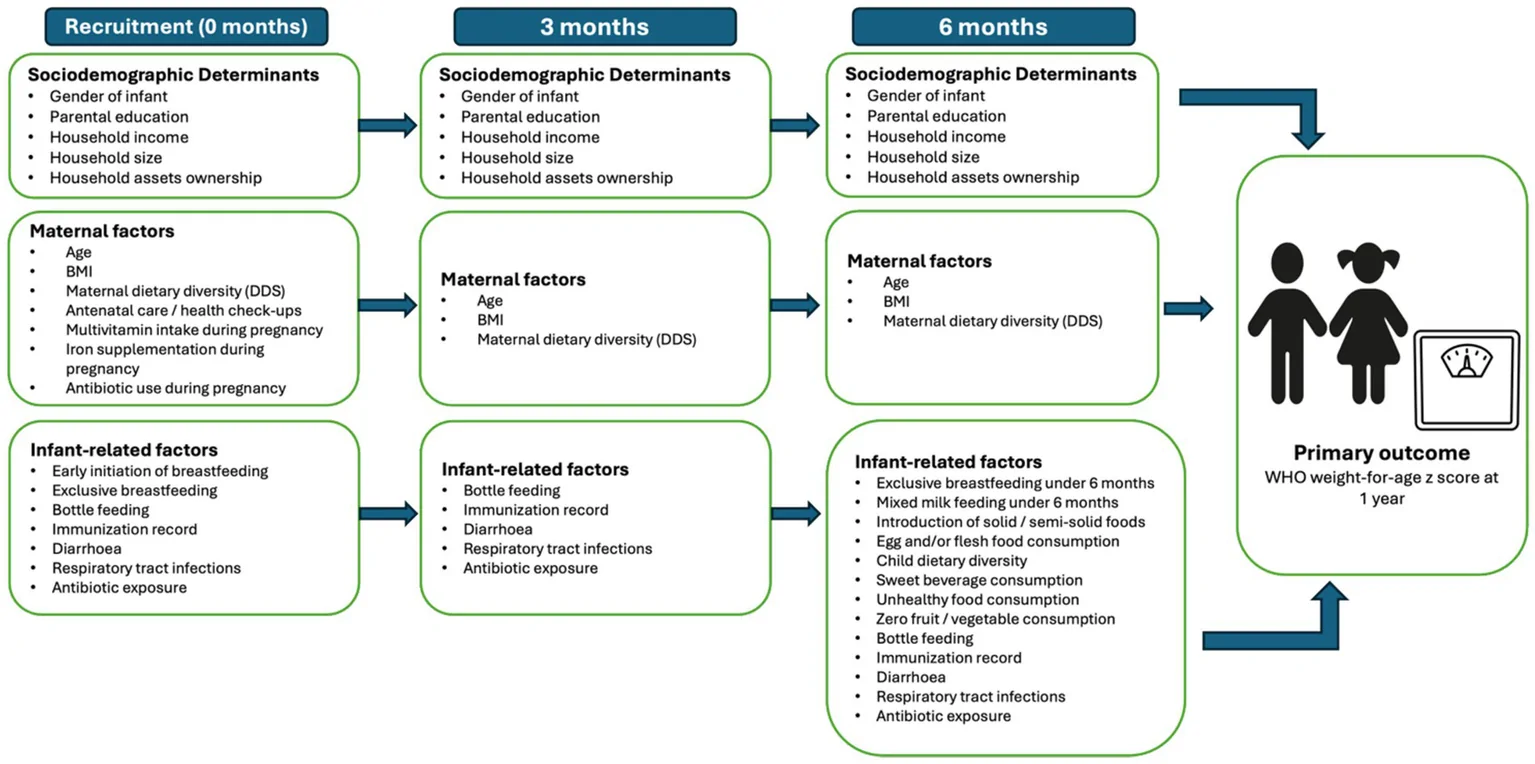
Conceptual framework of time-based determinants of infant growth CHAMP cohort, rural Pakistan.

We developed a time-staged conceptual framework (Figure 1) to highlight potential factors present during the first 6 months of life and that are known to be linked to child growth in Pakistan. The framework posits that sociodemographic characteristics, maternal factors and infant characteristics operating during the first 6 months of life jointly influence child growth. Based on this conceptual framework, variables were aligned in groups of possible predictors as three time-staged domains measured at recruitment (0–28 days), 3 months and 6 months.

### 2.3 Statistical analyses

All analyses were conducted using SPSS (IBM Corp., version 27) and RStudio (version 2025.09.2+418). Continuous variables were summarised using mean (SD) or median (IQR), and categorical variables using counts and percentages.

#### 2.3.1 Variable pre-selection and domain time-staged models

To reduce redundancy among feeding indicators, particularly at 6 months, Spearman rank correlations were used to assess overlap among binary/ordinal dietary measures and to guide parsimonious model specification. Supplementary material S1 shows moderate correlations among 6-month feeding indicators, indicating partial overlap between measures. As expected, exclusive breastfeeding <6 months was inversely correlated with mixed milk feeding and related to bottle feeding, reflecting a shared early feeding construct. Several complementary feeding indicators also clustered, with sweet beverage consumption correlating with multiple diet variables, suggesting redundancy if entered concurrently. Egg/flesh food consumption (EFF) showed weaker-to-moderate correlations with other measures, supporting its role as a relatively distinct marker of complementary feeding quality. These findings informed the parsimonious model specification by avoiding simultaneous inclusion of highly overlapping indicators.

We used linear regression to estimate associations with weight-for-age z-score (WAZ) at 1 year. To preserve temporal ordering and enhance interpretability, separate multivariable models were fitted for each of the four domains (sociodemographic, maternal and infant-related factors relating to diet and morbidity), retaining all variables within each domain at the corresponding time point. Across domain models, child sex was included as a core covariate, and maternal education was used as the primary socioeconomic adjustment variable to improve interpretability and limit redundancy across multiple SES indicators. Maternal education was selected as the primary socioeconomic status (SES) covariate, based on its consistent signal across domain models and its established use as a proxy for socioeconomic position.

Within the infant-related domain, feeding practices (exclusive breastfeeding duration, complementary feeding type) and morbidity (diarrhoea, respiratory tract infection, antibiotic use) were modelled in separate non-overlapping models to aid interpretation.

#### 2.3.2 Final model specification

The final etiologic model included child sex, maternal education (any education vs. no formal education), exclusive breastfeeding under 6 months (EBF) and egg/flesh food consumption measured at 6 months (EFF). This model was chosen based on the domain analysis (time-staged) results and the conceptual framework.

Given the fixed cohort size, we prioritised parsimonious model specification to preserve an adequate ratio of observations to predictors. The pre-specified final 6-month model contained four terms (child sex, maternal education, exclusive breastfeeding and egg/flesh food consumption), corresponding to approximately 16–17 observations per predictor at *n* = 66–70, within the conventional 10–15 observations per predictor threshold for stable estimation. The larger exploratory domain models carried 5–9 predictors (approximately 7–13 observations per predictor) and were therefore interpreted as screening analyses rather than definitive tests. For an *F*-test of the four-predictor final model (*n* = 66, *α* = 0.05), the study had approximately 80% power to detect an *R*^2^ of about 0.18 (f^2^ = 0.22, a medium effect); smaller effects were correspondingly under-powered, which is reflected in the wide confidence intervals reported for borderline associations. Because a single model containing all domain variables would have exceeded a defensible number of predictors for this sample size, the time-staged domain approach was adopted as a pre-specified dimensionality-reduction strategy grounded in the conceptual framework (Figure 1). This approach is grounded in Ockham’s Razor (Occam’s Razor) principle of parsimony; given comparable explanatory adequacy, the model with the fewest unnecessary parameters is preferred, guarding against overfitting at this sample size (23, 24).

#### 2.3.3 Model diagnostics and reporting

Model fit was assessed using *R*^2^ and *F*-tests for overall model significance. Multicollinearity was evaluated using variance inflation factors (VIF) and tolerance statistics. Results are reported as unstandardized regression coefficients (B) with 95% confidence intervals and two-tailed *p*-values. Statistical significance was set at *α* = 0.05.

#### 2.3.4 Sensitivity analyses

To assess the robustness of the primary findings, five prespecified sensitivity analyses were conducted:

1. Baseline adjustment. The final 6-month models were additionally adjusted for WAZ or HAZ at recruitment, as appropriate, so that estimates reflect associations with 12-month z-scores conditional on size at recruitment.
2. Joint (bivariate) model. WAZ and HAZ at 12 months were entered as simultaneous dependent variables in a multivariate linear model, allowing their errors to correlate, to accommodate the fact that the two outcomes are derived from the same infant and are not independent.
3. Repeated-measures model. A random-intercept linear mixed model used anthropometry from all four occasions (0, 3, 6 and 12 months) to test whether the maternal education association held when all measurement occasions were modelled jointly rather than at a single endpoint.
4. Leave-one-cluster-out analysis. The final models were refitted excluding the set of triplets, whose members are statistically non-independent, to assess the influence of this multiple-birth cluster.
5. Composite model. A parsimonious composite model carried forward the leading correlate from each domain (maternal education, the 6-month feeding indicators, and 6-month diarrhoea), mutually adjusted and adjusted for child sex, to estimate their combined contribution within the constraints of the available sample size.

All models were assessed for multicollinearity using variance inflation factors and tolerance statistics. Model fit was summarised using *R*^2^, and overall model significance assessed using the *F*-test. Results are reported as unstandardised coefficients (B) with 95% confidence intervals and *p*-values. Full specifications and results for analyses (2)–(5) are provided in the Supplementary material.

## 3 Results

### 3.1 Characteristics of the population

At baseline, 70 mothers and 72 newborn infants—including one set of triplets—were recruited from rural, mountainous communities in District Swat, Pakistan. Table 1 presents household, maternal and infant characteristics at recruitment, and at 3, 6 and 12 months postpartum, stratified by infant sex. Parental education levels were generally low: 67.1% of mothers and 51.4% of fathers had received no formal education. Most households were of lower socioeconomic background, with an average monthly household income of 18,857 PKR (~US$67) well below the national minimum wage (36,000 PKR) at the time of recruitment. Regarding maternal nutritional status, the majority (64.3%) had normal BMI. Maternal dietary diversity was poor, with a mean dietary diversity score (DDS) of 3, and no mother met the minimum DDS threshold for women (≥5 food groups). Only a quarter of the infants received exclusive breastfeeding during the first 2 days of life. Diarrhoea, respiratory tract infections (RTI) and antibiotic exposure were common, each reported in more than 40% of infants in the early postnatal period. At 3-month follow-up, the maternal DDS score remained low with high proportion of infants experiencing diarrhoea and RTIs. At 6 months, low dietary diversity (DDS 0–2) was observed in 69.7% of infants, with exclusive breastfeeding or mixed milk feeding in 43.1 and 25% of infants, respectively. Fruit and vegetables (22.7%), and eggs and flesh food (14.9%) consumption was also low. Child morbidity remained higher with high prevalence of diarrhoea (58.5%) and RTI (69.2%). At the 12-month follow-up, maternal and child DDS remained low, although consumption of fruit and vegetables (30%), and eggs and flesh foods (21%) had improved modestly. However, consumption of sweet beverages and unhealthy food was high being reported by 54.3 and 39.7%, respectively, of the participants. Overall, dietary diversity, and infant and child feeding practices remained suboptimal throughout the follow-up.

**Table 1:** Characteristics of the study population at baseline and at 3, 6 and 12 months postpartum (*n* = 72 infants from 70 mothers, including one triplet set).

Variable	Overall, *n*/*N* (%)
Baseline (0–28 days)	
Maternal age, years (mean ± SD)	27.8 ± 6.6
Monthly household income, PKR (mean ± SD)	18,857 ± 12,142
Mother’s literacy	
No formal education	47/70 (67.1%)
Any level of education	23/70 (32.9%)
Father’s literacy	
No formal education	36/70 (51.4%)
Any level of education	34/70 (48.6%)
Total assets	
≤3	43/70 (61.4%)
>3	27/70 (38.6%)
Maternal BMI	
Underweight	8/70 (11.4%)
Normal	45/70 (64.3%)
Overweight/obese	17/70 (24.3%)
Maternal DDS	
0–2	40/70 (57.1%)
>2	30/70 (42.9%)
Early initiation of breastfeeding (Yes)	16/72 (22.2%)
Exclusive breastfeeding during first 2 days (Yes)	18/72 (25.0%)
Bottle feeding (Yes)	20/72 (27.8%)
Child immunization record available (Yes)	29/71 (40.8%)
Diarrhoea (Yes)	34/72 (47.2%)
Respiratory tract infection (Yes)	30/72 (41.7%)
Antibiotic exposure (Yes)	29/71 (40.8%)
3 months	
Maternal DDS^*^	0–2: 40/70 (57.1%); >2: 30/70 (42.9%)
Bottle feeding (Yes)	19/64 (29.7%)
Diarrhoea (Yes)	58/63 (92.1%)
Respiratory tract infection (Yes)	51/64 (79.7%)
Antibiotic exposure (Yes)	56/64 (87.5%)
6 months	
Bottle feeding (Yes)	20/66 (30.3%)
Maternal DDS	0–2: 48/70 (68.6%); >2: 22/70 (31.4%)
Diarrhoea (Yes)	38/65 (58.5%)
Respiratory tract infection (Yes)	45/65 (69.2%)
Antibiotic exposure (Yes)	45/65 (69.2%)
Child DDS	0–2: 46/66 (69.7%); >2: 20/66 (30.3%)
Sweet beverage consumption^**^	≤2: 10/65 (15.4%); >2: 55/65 (84.6%)
Exclusive breastfeeding under 6 months (Yes)	31/72 (43.1%)
Mixed milk feeding under 6 months (Yes)	18/72 (25.0%)
Unhealthy food consumption (Yes)^***^	21/66 (31.8%)
Vegetable/fruit consumption (Yes)	15/66 (22.7%)
Egg/flesh food consumption (Yes)	10/67 (14.9%)
12 months	
Bottle feeding (Yes)	15/66 (22.7%)
Diarrhoea (Yes)	48/65 (73.8%)
Maternal DDS	0–2: 47/70 (67.1%); >2: 23/70 (32.9%)
Child DDS	0–2: 40/68 (58.8%); >2: 28/68 (41.2%)
Vegetable/fruit consumption (Yes)	20/66 (30.3%)
Egg/flesh food consumption (Yes)	14/66 (21.2%)
Sweet beverage consumption	≤2: 32/70 (45.7%); >2: 38/70 (54.3%)
Unhealthy food consumption (Yes)	27/68 (39.7%)

Data are presented as *n*/*N* (%) unless otherwise indicated. Continuous variables are presented as mean ± SD. For binary variables, only the ‘Yes’ category is shown; complementary ‘No’ values can be inferred from the reported denominators. *N* varies owing to missing data and loss to follow-up. The cohort comprised 72 infants born to 70 mothers, including one set of triplets; maternal characteristics are therefore reported at the mother level (*N* = 70) and infant characteristics at the child level (*N* = 72).

Dietary diversity score scores were assessed using country (Pakistan) specific Dietary Quality Questionnaire -DQQ for adults (Supplementary material S6) and DQQ for IYCF (Supplementary material S7).

^*^Maternal DDS at 3 months was recorded as unchanged from baseline for every participant in the analysis dataset.

^**^Sweet-beverage intake was recorded as the number of consumption occasions in the preceding 24 h (observed range 1–8 occasions); no child recorded zero occasions.

^***^Unhealthy food consumption is defined in accordance with the WHO infant and young child feeding (IYCF) indicator framework as consumption, in the preceding 24 h, of one or more energy-dense, nutrient-poor items discouraged under WHO IYCF guidance.

BMI, body mass index; DDS, dietary diversity score; EBF, exclusive breastfeeding; EBF2D, exclusive breastfeeding in the first 2 days; EFFA, egg and/or flesh-food consumption; EIBF, early initiation of breastfeeding; IYCF, infant and young child feeding; PKR, Pakistani Rupee; RTI, respiratory tract infection.

### 3.2 Prevalence of undernutrition

**Figure 2:**
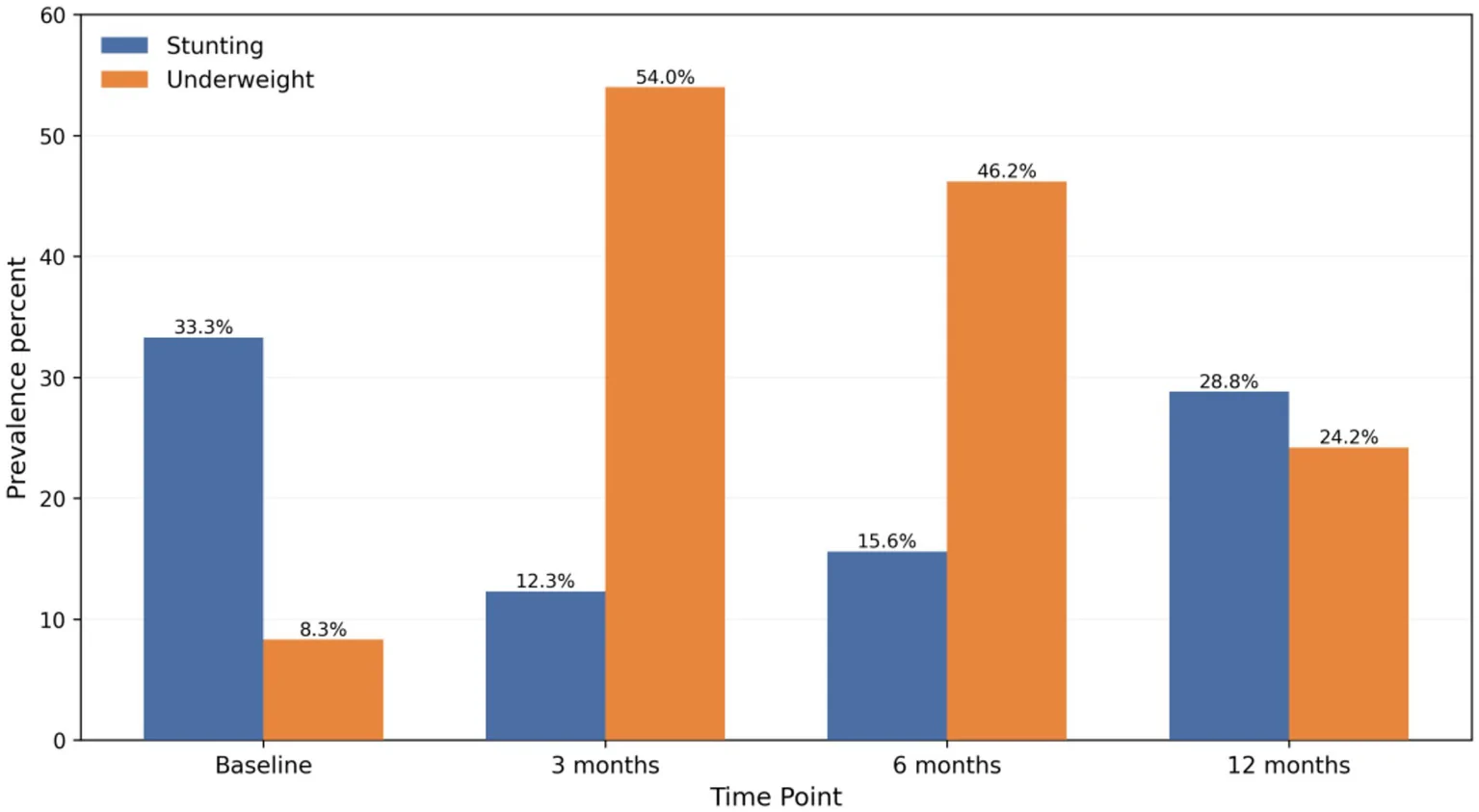
Prevalence of stunting and underweight across time points.

**Figure 3:**
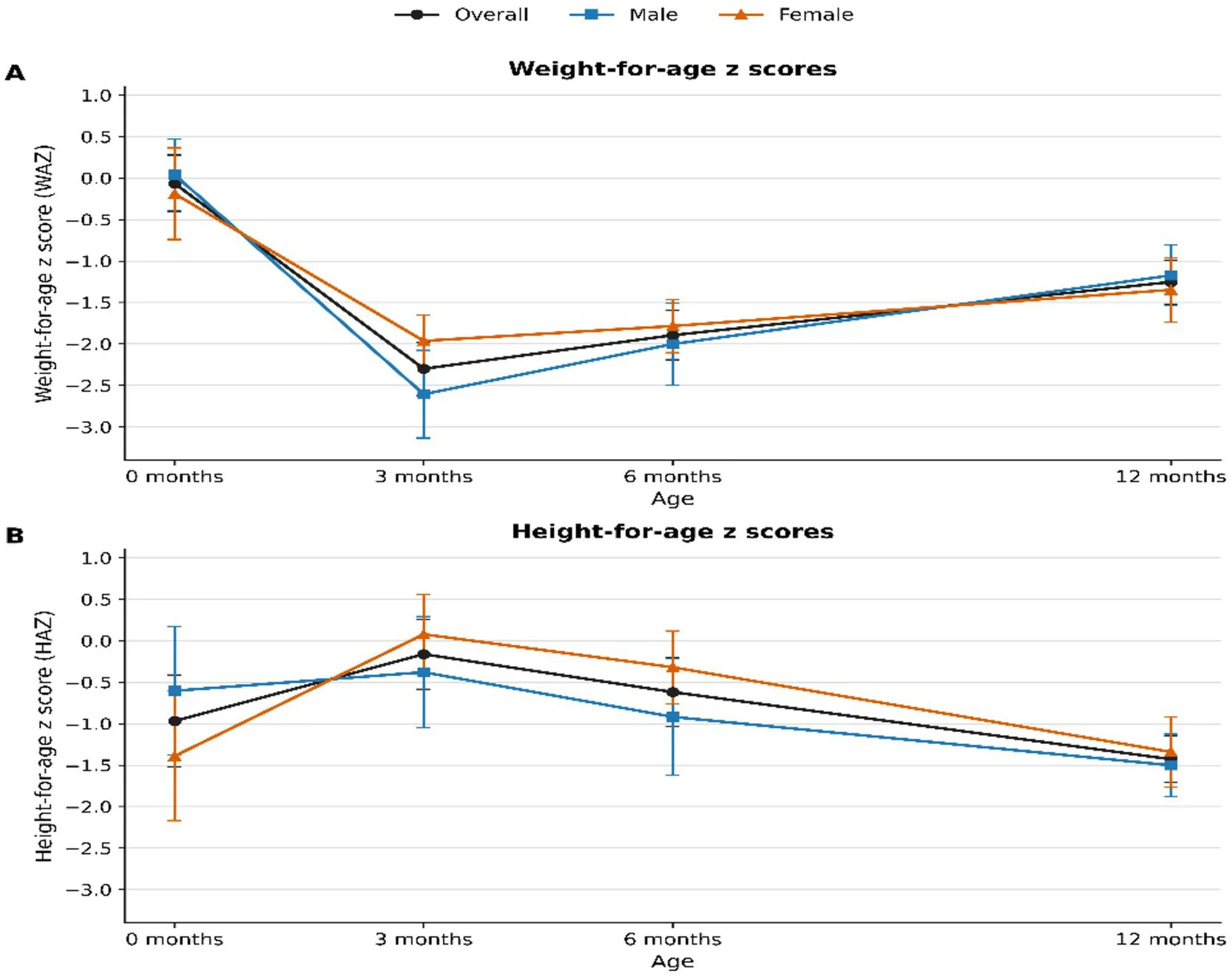
Growth trajectories of infants from baseline to 1 year based on WHO growth standards **(A)** Weight for age z-score—WAZ and **(B)** Height/length for age z-score – LAZ/HAZ.

Anthropometric assessment during the first year confirmed growth vulnerability in the study population. At recruitment (0–28 days), 8.3% of infants were classified as underweight (WAZ < −2 SD) and 33.3% as stunted (HAZ < −2 SD). Stunting prevalence then declined sharply at 3 months (12.3%) before rising progressively at 6 months (14.1%) and 12 months (28.8%), returning towards its baseline level. Underweight prevalence showed the opposite early pattern, rising to 53.8% at 3 months before declining to 46.2% at 6 months and 22.7% at 12 months (Figure 2). Figures 3A,B shows gender-based, growth trajectories of the infants from 0 to 12 months. The longitudinal WAZ trajectory demonstrated a marked decline between recruitment (0 months) and 3-months, followed by a gradual increase through six and 12 months; a pattern consistent with early growth faltering and incomplete catch-up growth (Figure 3A). In contrast, the mean height-for-age increased from baseline to 3-months followed by a gradual decrease at 6 and 12 months.

### 3.3 Early predictors of WAZ and HAZ at 12 months

#### 3.3.1 Socioeconomic and demographic predictors

The baseline socioeconomic model incorporating all measured socioeconomic and demographic indicators demonstrated limited ability to explain variations in WAZ and HAZ score at 12 months. Overall, the model explains only 10.6% of the variations in WAZ score (*R*^2^ = 0.106) and 12.9% in HAZ score (*R*^2^ = 0.129) and was not statistically significant overall (Table 2). Among individual predictors, maternal education showed the strongest and most consistent association with both WAZ (*B* = 0.685, 95% CI −0.117 to 1.487, *p* = 0.093) and HAZ (*B* = 0.815, 95% CI: −0.015 to 1.645, *p* = 0.054) at 12 months. Although this association does not reach statistical significance, the overall trend suggests a positive association between maternal education and infant growth (WAZ-score) at 12 months. Given its consistent directional signal and established role as a stable socioeconomic proxy in the literature, maternal education was retained as the primary SES adjustment covariate in all subsequent domain models.

**Table 2:** Associations of sociodemographic factors with weight for age (WAZ) and height for age z-score (HAZ) at 12-months.

Predictor	Weight for age z-score *n* = 64		Height for age z-score (HAZ) *n* = 64	
	*B* (95% CI)	*p*-value	*B* (95% CI)	*p*-value
Socio-demographic domain model	*R*^2^ = 0.106	Model *p* = 0.479	*R*^2^ = 0.129	Model *p* = 0.325
Child sex (Female)	−0.070 (−0.680, 0.541)	0.820	0.309 (−0.323, 0.941)	0.331
Child age (days)	−0.023 (0.060, 0.015)	0.226	−0.023 (−0.061, 0.016)	0.245
Paternal education	0.012 (−0.695, 0.719)	0.973	−0.07 (−0.802, 0.661)	0.848
Maternal education	0.685 (−0.117, 1.487)	0.093	0.815 (−0.015,to 1.645)	**0.054**
Household income (per 10,000 PKR increase)	−0.001 (−0.247, 0.246)	0.996	−0.007 (−0.261, 0.248)	0.958
Number of household members	−0.003 (−0.129, 0.123)	0.962	−0.007 (−0.137, 0.124)	0.92
Household assets score	−0.085 (−0.243, 0.072)	0.280	−0.115 (−0.277, 0.048)	0.163

*B*, unstandardised regression coefficient; CI, confidence interval; PKR, Pakistani Rupees. Household assets score reflects the sum of all recorded asset indicators. Outcome variable: WAZ and HAZ at 12 months. *n* denotes the number of infants contributing complete data on all variables in that model (complete-case analysis); *n* therefore varies across models owing to item-level missingness. Bold values indicate statistically significant *p*-values.

#### 3.3.2 Maternal predictors

The maternal domain model at baseline (recruitment), incorporating maternal age, pregnancy-related healthcare indicators, BMI and dietary diversity, showed modest explanatory power for both WAZ (*R*^2^ = 0.139) and HAZ (*R*^2^ = 0.174). The model was not statistically significant overall (Table 3). In this model, maternal education showed a borderline, significant positive association with WAZ at 12 months (*B* = 0.675, 95% CI −0.001 to 1.351, *p* = 0.050). In contrast, other maternal factors were not independently associated with WAZ and HAZ at 12 months.

**Table 3:** Associations of maternal factors at baseline, 3 and 6-months with weight for age (WAZ) and height for age z-score (HAZ) at 12-months.

Predictor	Weight for age z-score		Height for age z-score (HAZ)	
	*B* (95% CI)	*p*-value	*B* (95% CI)	*p*-value
At recruitment	*n* = 64 *R*^2^ = 0.139	Model *p* = 0.473	*n* = 64 *R*^2^ = 0.174	Model p = 0.276
Child sex (Female)	0.002 (−0.623 to 0.626)	0.996	0.185 (−0.440, 0.811)	0.555
Maternal education	0.675 (−0.001 to 1.351)	**0.05**	0.718 (−0.052, 1.488)	0.067
Maternal age (years)	0.007 (−0.038 to 0.053)	0.745	−0.016 (−0.065, 0.032)	0.509
Maternal BMI	0.046 (−0.037 to 0.130)	0.27	−0.021 (−0.107, 0.065)	0.624
Maternal dietary diversity score (DDS)	−0.116 (−0.423 to 0.192)	0.454	0.026 (−0.287, 0.338)	0.87
Number of ANC visits	−0.053 (−0.173 to 0.067)	0.379	0.045 (−0.075, 0.165)	0.454
Multivitamin use during pregnancy	0.120 (−0.726 to 0.966)	0.777	0.366 (−0.449, 1.182)	0.372
Iron supplementation during pregnancy	−0.432 (−1.286 to 0.422)	0.315	−0.590 (−1.418, 0.238)	0.159
Antibiotic use during pregnancy	0.290 (−0.337 to 0.917)	0.358	−0.327 (−1.009, 0.356)	0.342

Predictor	Weight for age z-score		Height for age z-score (HAZ)	
	*B* (95% CI)	*p*-value	*B* (95% CI)	*p*-value
Three months postpartum	*n* = 59 *R*^2^ = 0.085	Model *p* = 0.297	*n* = 59 *R*^2^ = 0.079	Model *p* = 0.338
Child sex (Female)	−0.119 (−0.734 to 0.496)	0.699	0.268 (−0.348, 0.884)	0.386
Maternal education	0.615 (−0.060 to 1.289)	0.073	0.709 (0.032, 1.385)	**0.04**
Maternal BMI at 3 months	0.034 (−0.045 to 0.114)	0.392	0.015 (−0.065, 0.094)	0.715
Maternal DDS at 3 months	−0.060 (−0.373 to 0.254)	0.705	−0.066 (−0.379, 0.248)	0.677

Predictor	Weight for age z-score		Height for age z-score (HAZ)	
	*B* (95% CI)	*p*-value	*B* (95% CI)	*p*-value
Six months postpartum	*n* = 59 *R*^2^ = 0.074	Model *p* = 0.375	*n* = 59 *R*^2^ = 0.082	Model *p* = 0.321
Child sex (Female)	−0.006 (−0.655 to 0.643)	0.985	0.303 (−0.358, 0.963)	0.362
Maternal education	0.706 (−0.035 to 1.448)	0.062	0.606 (−0.149, 1.361)	0.113
Maternal BMI at 6 months	−0.004 (−0.080 to 0.072)	0.917	0.017 (−0.060, 0.094)	0.661
Maternal DDS at 6 months	−0.049 (−0.266 to 0.168)	0.653	0.066 (−0.156, 0.287)	0.554

B, unstandardised regression coefficient; CI, confidence interval; DDS, Dietary Diversity Score; BMI, body mass index; ANC, antenatal care. Each time-point block represents a separate regression model. Outcome variable: WAZ and HAZ at 12 months. All models adjusted for child sex and maternal education. *n* denotes the number of infants contributing complete data on all variables in that model (complete-case analysis); *n* therefore varies across models owing to item-level missingness. Bold values indicate statistically significant *p*-values.

Maternal domain models at 3 and 6-months similarly showed limited explanatory capacity for WAZ and HAZ and were not statistically significant overall. However, maternal education at 3 and 6 months consistently showed a positive association with WAZ and HAZ at 12-months. The consistent directional trend across all three time points suggests a potential influence of maternal education on infant growth despite not reaching conventional thresholds of statistical significance.

#### 3.3.3 Infant related morbidity predictors

Infant morbidity indicators including diarrhoeal episodes, respiratory tract infection (RTI) and antibiotic exposure at baseline, 3 months and 6 months were not independently associated with WAZ and HAZ at 12 months (Table 4). The overall morbidity model showed poor explanatory power and was not statistically significant with WAZ and HAZ at 12-months. Among individual factors, only maternal education emerged as a significant positive predictor of WAZ (*B* = 0.862, 95% CI 0.158–1.566, *p* = 0.017) as well as HAZ (*B* = 0.772, 95% CI 0.023, 1.520, *p* = 0.043) at 12 months. This represents the only statistically significant individual predictor across the three-morbidity time-point models and reinforces the consistent pattern observed in sociodemographic and maternal analysis.

**Table 4:** Associations of infant related morbidity factors at baseline, 3 and 6-months with weight for age (WAZ) and height for age z-score (HAZ) at 12-months.

Predictor	Weight for age z-score		Height for age z-score (HAZ)	
	*B* (95% CI)	*p*-value	*B* (95% CI)	*p*-value
At Recruitment	*n* = 65 *R*^2^ = 0.08	Model *p* = 0.401	*n* = 65 *R*^2^ = 0.102	Model *p* = 0.261
Child sex (Female)	−0.065 (−0.662 to 0.531)	0.827	0.223 (−0.393, 0.840)	0.472
Maternal education	0.635 (−0.009 to 1.279)	0.053	0.651 (−0.015, 1.317)	0.055
Diarrhoea	0.089 (−0.481 to 0.658)	0.757	0.045 (−0.544, 0.633)	0.88
Respiratory tract infection (RTI)	0.045 (−0.550 to 0.640)	0.881	0.401 (−0.214, 1.016)	0.197
Antibiotic exposure	−0.292 (−0.902 to 0.318)	0.342	−0.372 (−1.002, 0.259)	0.243

Predictor	Weight for age z-score		Height for age z-score (HAZ)	
	*B* (95% CI)	*p*-value	*B* (95% CI)	*p*-value
Three months	*n* = 60 *R*^2^ = 0.144	Model *p* = 0.126	*n* = 60 *R*^2^ = 0.077	Model *p* = 0.486
Child sex (Female)	−0.012 (−0.646 to 0.622)	0.969	0.367 (−0.307, 1.041)	0.28
Maternal education	0.862 (0.158 to 1.566)	**0.017**	0.772 (0.023, 1.520)	**0.043**
Diarrhoea	0.342 (−0.728 to 1.412)	0.524	−0.063 (−1.200, 1.074)	0.912
RTI	−0.052 (−0.866 to 0.763)	0.899	0.199 (−0.667, 1.065)	0.647
Antibiotic exposure	−0.751 (−1.782 to 0.281)	0.15	−0.281 (−1.378, 0.815)	0.609

Predictor	Weight for age z-score		Height for age z-score (HAZ)	
	*B* (95% CI)	*p*-value	*B* (95% CI)	*p*-value
Six months	*n* = 63 *R*^2^ = 0.072	Model *p* = 0.500	*n* = 63 *R*^2^ = 0.102	Model *p* = 0.278
Child sex (Female)	−0.025 (−0.613 to 0.563)	0.932	0.244 (−0.365, 0.853)	0.426
Maternal education	0.412 (−0.271 to 1.095)	0.232	0.597 (−0.110, 1.304)	0.096
Diarrhoea	0.362 (−0.238 to 0.963)	0.232	0.405 (−0.217, 1.027)	0.197
RTI	−0.056 (−0.843 to 0.730)	0.886	−0.014 (−0.828, 0.801)	0.973
Antibiotic exposure	−0.109 (−0.921 to 0.703)	0.789	−0.168 (−1.009, 0.673)	0.69

B, unstandardised regression coefficient; CI, confidence interval; RTI, Respiratory Tract Infection. ^*^p < 0.05. Each section represents a separate regression model. Outcome variable: WAZ and HAZ at 12 months. All models adjusted for child sex and maternal education. *n* denotes the number of infants contributing complete data on all variables in that model (complete-case analysis); *n* therefore varies across models owing to item-level missingness. Bold values indicate statistically significant *p*-values.

#### 3.3.4 Infant related feeding predictors

Regression models examining early-life feeding practices, assessed using the validated IYCF questionnaire, also showed limited explanatory power for child growth outcomes at both baseline and 3 months (Table 5). At recruitment, individual early feeding indicators such as early initiation of breastfeeding, exclusive breastfeeding in the first 2 days or bottle feeding were not independently associated with WAZ or HAZ at 12 months. However, maternal education was significantly associated with a higher HAZ (*B* = 0.666, 95% CI: 0.013–1.320, *p* = 0.046). A positive, but non-significant association between maternal education and WAZ was also observed (*B* = 0.595, 95% CI −0.031 to 1.220, *p* = 0.062). At 3-months, none of the model and individual factors were significantly associated with WAZ or HAZ at 12 months. In contrast, the 6-month complementary feeding model, incorporating egg/flesh food consumption (EFF), exclusive breastfeeding at under 6 months (EBF), maternal education and child sex, was the only feeding model to reach overall statistical significance for both outcomes, explaining 15.9% of the variation in WAZ (*R*^2^ = 0.159, *p* = 0.030) and 15.7% of the variation in HAZ (*R*^2^ = 0.157, *p* = 0.032; *n* = 66).

**Table 5:** Associations of child feeding and complementary feeding factors with WAZ and HAZ at 12 months, including the pre-specified final 6-month etiologic model—CHAMP Cohort.

Predictor	Weight for age z-score		Height for age z-score (HAZ)	
	*B* (95% CI)	*p*-value	*B* (95% CI)	*p*-value
At recruitment	*n* = 66 *R*^2^ = 0.101	Model *p* = 0.371	*n* = 66 *R*^2^ = 0.114	Model *p* = 0.192
Child sex (Female)	0.015 (−0.549 to 0.580)	0.957	0.371 (−0.219, 0.960)	0.213
Maternal education	0.595 (−0.031 to 1.220)	0.062	0.666 (0.013, 1.320)	0.046
Bottle feeding	0.266 (−0.380 to 0.912)	0.413	0.344 (−0.331, 1.019)	0.313
Early initiation of breastfeeding	−0.148 (−0.841 to 0.546)	0.672	−0.252 (−0.977, 0.472)	0.489
Exclusive breastfeeding (first 2 days)	0.188 (−0.507 to 0.884)	0.59	0.115 (−0.611, 0.842)	0.752

Predictor	Weight for age z-score		Height for age z-score (HAZ)	
	*B* (95% CI)	*p*-value	*B* (95% CI)	*p*-value
Three months	*n* = 61 *R*^2^ = 0.097	Model *p* = 0.118	*n* = 61 *R*^2^ = 0.097	Model *p* = 0.119
Child sex (female)	−0.036 (−0.641 to 0.570)	0.907	0.381 (−0.236, 0.998)	0.221
Maternal education	0.654 (−0.019 to 1.327)	0.056	0.670 (−0.016, 1.356)	0.055
Bottle feeding (3 months)	0.349 (−0.262 to 0.959)	0.257	0.417 (−0.205, 1.039)	0.185

Predictor	Weight for age z-score		Height for age z-score (HAZ)	
	*B* (95% CI)	*p*-value	*B* (95% CI)	*p*-value
Six months	*n* = 66 *R*^2^ = 0.159	Model *p* = 0.030	*n* = 66 *R*^2^ = 0.157	Model *p* = 0.032
Child sex (female)	−0.008 (−0.550 to 0.533)	0.975	0.326 (−0.245, 0.897)	0.258
Maternal education	0.528 (−0.083 to 1.139)	0.089	0.676 (0.033, 1.320)	**0.04**
Egg/flesh food consumption (EFF) 6–23 months	0.550 (−0.106 to 1.205)	0.099	0.208 (−0.483, 0.899)	0.549
Exclusive breastfeeding <6 months (EBF)	−0.417 (−0.942 to 0.108)	0.118	−0.613 (−1.166, −0.060)	**0.03**

B, unstandardised regression coefficient; CI, confidence interval; EBF, exclusive breastfeeding under 6 months. Each section represents a separate regression model. Outcome variable: WAZ and HAZ at 12 months. *n* denotes the number of infants contributing complete data on all variables in that model (complete-case analysis); *n* therefore varies across models owing to item-level missingness. Bold values indicate statistically significant *p*-values.

Among individual factors, exclusive breastfeeding for less than 6 months was significantly associated with lower HAZ (*B* = −0.613, 95% CI: −1.166 to −0.060, *p* = 0.030), suggesting poorer linear growth among children who were not exclusively breastfed for the recommended duration. A borderline positive association with WAZ at 12 months was also observed for eggs and/or flesh food consumption (*B* = 0.550, 95% CI −0.106 to 1.205, *p* = 0.099) (Table 5).

#### 3.3.5 Sensitivity analyses

We assessed the robustness of the 12-month findings in four ways: adjustment for baseline size, joint estimation of both outcomes, use of all four measurement occasions and exclusion of the triplet cluster. Full results are provided in Supplementary material.

Adjusting for anthropometry at recruitment (Table 6), both models remained significant (WAZ: *R*^2^ = 0.190, *p* = 0.024, *n* = 66; HAZ: *R*^2^ = 0.226, *p* = 0.011, *n* = 63). Baseline HAZ independently predicted 12-month HAZ (*B* = 0.155, 95% CI 0.029–0.282, *p* = 0.017), whereas baseline WAZ was not a significant predictor of 12-month WAZ (*B* = 0.137, 95% CI −0.042 to 0.317, *p* = 0.131). After baseline adjustment, no feeding or maternal predictor retained significance in either model; the exclusive breastfeeding-HAZ association was attenuated to non-significance (*B* = −0.410, 95% CI −0.979 to 0.160, *p* = 0.155) (Table 6).

**Table 6:** Sensitivity analysis of predictors of weight-for-age and height-for-age z-scores.

Predictor	Weight for age z-score (WAZ) (*n* = 66)		Height for age z-score (HAZ) (*n* = 63)	
	*B* (95% CI)	*p*-value	*B* (95% CI)	*p*-value
Sensitivity analysis model	*R*^2^ = 0.190	Model *p* = 0.024	*R*^2^ = 0.226	Model *p* = 0.011
Child sex (Female)	0.035 (−0.504 to 0.574)	0.897	0.426 (−0.151, 1.004)	0.145
Mother education	0.465 (−0.145 to 1.075)	0.133	0.475 (−0.192, 1.142)	0.16
Egg and/or flesh food consumption (yes)	0.562 (−0.087 to 1.211)	0.088	0.126 (−0.574, 0.826)	0.719
Exclusive breastfeeding under 6 months	−0.316 (−0.852 to 0.221)	0.244	−0.410 (−0.979, 0.160)	0.155
Baseline WAZ at recruitment	0.137 (−0.042 to 0.317)	0.131	–	–
Baseline HAZ at recruitment	–	–	0.155 (0.029, 0.282)	0.017

WAZ and HAZ were strongly correlated at 12 months (Pearson *r* = 0.697, *p* < 0.001; *n* = 66) and remained so after adjustment (residual *r* = 0.665). Estimating both outcomes jointly left the regression coefficients unchanged, but no predictor reached *α* = 0.05 in the joint tests; exclusive breastfeeding was closest to the threshold (*p* = 0.097) (Supplementary material S2).

Using all four measurement occasions, between-child clustering was substantial for HAZ (ICC = 0.38) but minimal for WAZ (ICC = 0.04). Maternal education was associated with higher HAZ averaged across infancy (*B* = 1.30, 95% CI 0.60–1.99, *p* < 0.001), in the same direction as the 12-month endpoint model; no corresponding association was observed for WAZ (*B* = 0.25, 95% CI −0.20 to 0.69, *p* = 0.285) (Supplementary material S3).

Excluding the set of triplets left the direction and magnitude of the 6-month feeding associations essentially unchanged, although the exclusive breastfeeding-HAZ association became borderline at the reduced sample size (*p* = 0.030 to *p* = 0.063; *n* = 66–63) (Supplementary material S4).

In a parsimonious composite model mutually adjusting maternal education, exclusive breastfeeding, 6-month egg and/or flesh food consumption and 6-month diarrhoea for child sex, the direction of these associations was preserved, though confidence intervals remained wide (Supplementary material S5).

## 4 Discussion

This prospective cohort study provides rare longitudinal evidence on early growth trajectories among infants living in remote rural communities of Swat. The results showed that WAZ markedly declined at age 1–3 months and recovery afterwards, while HAZ, which was improving initially obvious decline at the ages of 6–12 months. Maternal education emerged as the consistent direct correlate of growth, presenting positive associations with anthropometric indicators across all four models. Similarly, feeding practices overall and exclusive breastfeeding specifically showed satisfactory significance to growth. These findings should be interpreted alongside our previously published CHAMP cohort profile paper, which documented severe baseline socioeconomic vulnerability, poor maternal dietary diversity, inadequate antenatal counselling, suboptimal breastfeeding practices and early growth deficits in the same population (21). The present study extends that work by examining which early vulnerabilities persist, intensify or emerge as correlates of later infant growth.

Firstly, growth vulnerability during the first year of life appeared dynamic rather than linear. Underweight prevalence declined over the follow up period, while the mean WAZ trajectory showed an early decline followed by partial recovery. In contrast, mean HAZ improved initially after recruitment but declined again by 6 and 12 months, suggesting that linear growth vulnerability re-emerged later in infancy. These shifting anthropometric trajectories occurred within a broader context of persistent nutritional vulnerability. Maternal dietary diversity remained consistently poor across all follow up periods, early breastfeeding practices were suboptimal and complementary feeding quality remained inadequate. By 12 months, nearly 58.8% of infants consumed diets limited to more than 2 food groups, while over half consumed sweet beverages and almost 40% consumed unhealthy foods. This pattern is supported by longitudinal and regional evidence showing that infant growth faltering is not a simple linear process. Large pooled LMIC cohort analyses demonstrate that growth faltering often begins early in infancy and that early deficits increase the risk of later persistent growth failure (25). This may help explain why ponderal growth may deteriorate early and then partially recover, while linear growth deficits may re-emerge later as cumulative nutritional and environmental exposures intensify. Evidence from the MAL ED birth cohort further supports the role of dietary intake, illness and enteric exposures in shaping growth during the first 2 years of life (19). The nutritional vulnerability observed in this cohort is also consistent with Pakistan specific evidence. National PDHS based analysis found very low achievement of minimum dietary diversity and minimum acceptable diet among children aged 6–23 months (26). Similar patterns have been reported in Pakistan, where even children from middle socioeconomic households experienced poor dietary diversity and routine consumption of sucrose added foods and soft drinks (27). Taken together, these findings suggest that later linear growth vulnerability in this cohort may be partly shaped by poor complementary feeding quality, limited dietary diversity and early exposure to energy dense but nutrient poor foods. In the Pakistani context, these findings highlight the need to strengthen infant nutrition counselling through the Lady Health Worker Programme and existing maternal and child nutrition initiatives such as the Benazir Nashonuma Programme (28, 29). Counselling should place greater emphasis on maternal nutrition, exclusive breastfeeding support, culturally appropriate complementary feeding education and reducing children’s exposure to unhealthy commercial foods. Importantly, these interventions should not be limited to generic messages, but should include practical household level guidance on affordable, locally available and nutrient dense foods.

Secondly, conventional socioeconomic indicators, including household income, household assets, household size and paternal education, showed limited explanatory value for infant growth outcomes in this uniformly low-income cohort. In contrast, maternal education emerged as the most consistent upstream correlate across socioeconomic, maternal, morbidity and feeding models, demonstrating repeated positive associations with both WAZ and HAZ, although several estimates were borderline and should be interpreted cautiously. This pattern suggests that maternal education may influence infant growth through pathways beyond household economic resources alone, potentially including health literacy, caregiving practices, healthcare engagement and maternal decision-making capacity. These findings are broadly consistent with prior literature in which maternal education has emerged as a critical determinant of child nutritional outcomes, often operating through pathways that extend beyond household economic resources (6). Evidence from a large longitudinal cohort analysis across five low- and middle-income countries showed that maternal characteristics and early-life nutritional conditions have profound implications for subsequent growth trajectories, educational attainment, and long-term human capital outcomes. These findings highlight the intergenerational consequences of early nutritional disadvantage (6). Similarly, a large cross sectional analysis of over 900,000 households in Indonesia and Bangladesh reported that both maternal and paternal education were independently associated with lower risks of childhood stunting, partly through improved caregiving behaviours, immunization uptake and household health practices (30). While our findings differ in that paternal education was not significantly associated with infant growth outcomes, this discrepancy may reflect the relatively homogeneous socioeconomic conditions of our cohort, where limited variation in household resources may have reduced the explanatory value of conventional socioeconomic indicators. This interpretation is further supported by a multi-country analysis of 121 Demographic and Health Surveys across 36 LMICs, which found that economic growth alone was associated with only modest reductions in childhood undernutrition and conferred minimal benefit on the poorest households (31). Furthermore, evidence from a cross sectional analysis of Demographic and Health Survey data from Malawi, Tanzania and Zimbabwe demonstrated that higher maternal education was consistently associated with lower risks of stunting, wasting and underweight through improved health knowledge, feeding practices and healthcare mobilization (32). Collectively, these findings suggest that in settings characterised by widespread poverty, maternal education may represent a more proximate marker of infant growth vulnerability by influencing caregiving behaviours and the effective utilization of limited household resources. From a policy and practice perspective, low maternal education should be treated as an actionable risk marker rather than as a fixed background characteristic. In rural Pakistan, this could be addressed by strengthening existing community platforms rather than developing parallel systems. Scalable peer led community nutrition approaches such as the Care Group model have been used in LMIC settings to improve infant and young child feeding, dietary diversity and related nutrition behaviours (33–35). Adaptation of such approaches within the Lady Health Worker Programme and the Benazir Nashonuma Programme could combine low literacy counselling, practical demonstrations using affordable local foods, family inclusive engagement, early identification of growth faltering and referral linkages to primary healthcare services.

These associations are most plausibly understood within a hierarchical pathway in which distal socioeconomic conditions act upstream of maternal education and infant feeding practices, which in turn influence infant morbidity and growth. Consistent with partial mediation through feeding, the maternal education coefficient was attenuated when the 6-month feeding variables were introduced (from *B* = 0.68 in the sociodemographic model to *B* = 0.47 in the baseline-adjusted feeding model). We interpret this pathway cautiously; a formal structural equation or mediation model would require a substantially larger sample for stable path estimation.

Thirdly, our findings highlight the transition to complementary feeding at 6 months as a key modifiable window for later growth outcomes in this cohort. Unlike feeding models at recruitment and 3 months, the 6-month feeding model was the only feeding model to demonstrate overall statistical significance for both WAZ and HAZ. Within this model, failure to maintain exclusive breastfeeding for 6 months was associated with poorer linear growth, while egg and/or flesh food consumption showed a positive but borderline association with higher WAZ at 12 months. These findings should be interpreted cautiously given the modest sample size, but they identify the 6-month transition as an important point for intervention.

These findings are broadly consistent with global evidence identifying the period around 6 months as a critical transition point in infant nutrition, when breastmilk alone is no longer sufficient to meet all nutritional requirements and complementary foods should provide adequate dietary diversity, including energy and micronutrient rich foods, while breastfeeding continues (36). WHO guidance similarly recommends the introduction of complementary foods at 6 months and improving dietary diversity, including animal source foods such as eggs, meat and fish, for children aged 6–23 months (37). Evidence from Malawi also supports the association between exclusive breastfeeding during the first 6 months and improved later linear growth (38). However, exclusive breastfeeding should not be interpreted as a matter of maternal choice alone. Systematic review evidence indicates that it is shaped by intersecting maternal, socioeconomic, healthcare and support related factors, including breastfeeding awareness, family support, early initiation, maternal education, household income, antenatal care and breastfeeding self-efficacy (39). The positive but borderline association between egg and/or flesh food consumption and WAZ in our study is also consistent with meta analytic evidence showing modest improvements in weight and length related growth outcomes following animal source food supplementation in LMICs (40). However, individual trials have produced mixed findings. Egg supplementation improved growth in Ecuador (41), showed no overall effect on LAZ or WAZ in Malawi (42), and more recently appeared to improve ponderal rather than linear growth in Bangladesh (43). This mixed evidence suggests that animal source foods may support growth, but their effects are likely to depend on baseline nutritional status, infection burden, household food security, affordability, cultural acceptability and the extent to which they displace or complement other nutrient dense foods. Although national guidance in Pakistan recommends introducing safe and nutritionally adequate complementary foods at 6 months while continuing breastfeeding, our findings suggest that this guidance is not being translated into adequate household practice. Poor dietary diversity, limited consumption of animal source foods and early exposure to sweet beverages and unhealthy foods indicate the need for scalable, behaviour change focused complementary feeding support. Evidence from large scale infant and young child feeding programs in Bangladesh, particularly the Alive and Thrive model, shows that intensive interpersonal counselling, community mobilization and mass media delivered through existing health systems can improve complementary feeding practices at population level (44). In Pakistan, this model may be adapted by establishing the 6-month contact as a structured nutrition counselling touchpoint within Lady Health Worker visits, immunization contacts, and primary healthcare services. This should be supported by simple visual tools for low-literacy households and by family-inclusive counselling that involves fathers and grandmothers. Such an approach would directly address the observed gap between national complementary feeding guidance and household feeding practices by combining timely counselling, family support and practical food-based messages at the point when infant growth trajectories appear most modifiable.

The robustness of these feeding findings warrants careful appraisal. Although not maintaining exclusive breastfeeding to 6 months was associated with lower HAZ in the primary model (*B* = −0.613, 95% CI −1.166 to −0.060, *p* = 0.030), this association was attenuated and no longer statistically significant after adjustment for baseline height-for-age (*B* = −0.410, *p* = 0.155), and the egg/flesh food association with WAZ likewise weakened (*p* = 0.088). Two interpretations are possible and cannot be distinguished within this design. First, the attenuation may reflect partial confounding by initial size: infants who were shorter at recruitment may both have been fed differently and have grown less well, so that part of the apparent breastfeeding association reflects baseline status. Second, because baseline height-for-age is itself an early product of the same social and feeding environment, it may lie on the causal pathway, in which case adjusting for it constitutes over-adjustment and would be expected to attenuate a genuine association. We therefore regard the exclusive breastfeeding association as suggestive rather than robust, and give greater interpretive weight to the egg/flesh food direction, which, although non-significant, was preserved after baseline adjustment. That the overall model fit improved with baseline adjustment (HAZ *R*^2^ 0.157–0.226) indicates that baseline size carries substantial independent information about later growth.

A further finding concerns the differential tracking of the two growth dimensions. Baseline height-for-age significantly predicted 12-month HAZ (*B* = 0.155, 95% CI 0.029 to 0.282, *p* = 0.017), whereas baseline weight-for-age did not predict 12-month WAZ (*B* = 0.137, 95% CI −0.042 to 0.317, *p* = 0.131). This asymmetry indicates that linear growth trajectories were comparatively established early and tracked strongly through infancy, while ponderal growth remained more plastic. The pattern is consistent with the growth-faltering literature, in which stunting has its highest incidence of onset in the first months of life and is rarely reversed thereafter (45, 46), whereas weight-for-age responds more rapidly to proximate nutritional change, with measurable gains within weeks of supplementation (47). It also carries a practical implication: improvements in complementary feeding may yield weight-related gains within infancy, whereas meaningful gains in linear growth are likely to require earlier antenatal and early-infancy intervention.

This study has several strengths. It provides rare prospective evidence on infant growth trajectories from remote rural communities in Swat, Pakistan, a setting where longitudinal evidence on early growth faltering is limited and where most available evidence is derived from cross sectional national surveys. The repeated follow up design enabled assessment of how anthropometric status, maternal characteristics, morbidity and feeding related exposures evolved across the first year of life, allowing growth vulnerability to be examined longitudinally rather than inferred from a single time point. The use of standardised anthropometric indicators further strengthens the assessment of infant growth outcomes. The domain based and time staged analytical framework added further value by examining predictors across biologically and programmatically relevant periods of infancy, including recruitment, early infancy and the transition to complementary feeding. This approach is particularly relevant because the findings suggest that the 6-month feeding window may represent a key point for intervention. The study also adds context specific evidence from a geographically isolated and nutritionally vulnerable population, with direct relevance for rural nutrition policy and programme design in Pakistan.

This study is not without limitations. First and most importantly, the modest sample size (70 dyads) reduced statistical power, widened confidence intervals and constrained model complexity; the larger domain models carried fewer observations per predictor than is conventionally recommended and should be read as exploratory, and a single fully adjusted model, a formal mediation or structural equation model and a full growth trajectory (mixed-effects or GEE) analysis were not statistically credible at this sample size. Second, the cohort included one set of triplets whose members are statistically non-independent; a leave-one-cluster-out analysis indicated that the triplets did not drive the findings, but residual clustering cannot be excluded. Third, because two correlated outcomes (WAZ and HAZ) were examined, the single nominally significant individual association would not survive a Bonferroni-type adjustment for multiplicity, reinforcing a cautious interpretation.

The observational design also precludes causal inference and the associations should therefore be interpreted as hypothesis generating. Although repeated follow up was undertaken, morbidity was measured at discrete time points and may not have fully captured cumulative illness burden, episode duration, severity, recurrence or nutritional recovery following illness. Dietary and feeding variables may be affected by recall and social desirability bias, particularly in relation to breastfeeding and complementary feeding practices. In addition, the study did not directly measure maternal health literacy, caregiving agency, household decision making, food insecurity or intrahousehold food allocation, all of which may help explain the pathways linking maternal education with infant growth. Finally, as the cohort was drawn from remote rural communities in Swat, the findings may not be directly generalizable to urban populations or to other regions of Pakistan with different socioeconomic, cultural, dietary and health system contexts. Nevertheless, the longitudinal design, repeated growth measurements and focus on an underserved rural population provide valuable insight into early growth vulnerability in a setting where such evidence is scarce.

## 5 Conclusion

This longitudinal cohort study describes growth vulnerability among infants during the first year of life in remote rural communities of District Swat, Pakistan. Growth faltering in this cohort was dynamic rather than progressive, with weight-for-age declining early and partially recovering, while height-for-age deteriorated later in infancy. Conventional socioeconomic indicators and maternal dietary measures showed limited association with growth. In contrast, maternal education was the most consistent correlate of both outcomes and the 6-month feeding transition emerged as the period most closely linked to later growth: failure to maintain exclusive breastfeeding to 6 months was associated with lower height-for-age and consumption of animal-source foods showed a positive but borderline association with weight-for-age. These associations are hypothesis-generating and the exclusive breastfeeding finding was attenuated after adjustment for baseline size. Strengthening existing community-based programs may offer cost effective and culturally relevant approach to improve growth and development in infants during this critical period.

## Data Availability

The raw data supporting the conclusions of this article will be made available by the authors, without undue reservation.
