# Supplementary material for: Predictors of impaired growth during the first year of life: findings from a longitudinal cohort study in Pakistan
Source: Front Nutr. 2026 Aug 17;13:1912329. doi: 10.3389/fnut.2026.1912329 (PMC13526578; doi:10.3389/fnut.2026.1912329)
Supplement: Supplementary file 1 [file Table_1.docx]

**Supplementary Material S1. Correlations among 6-month feeding indicators**

The six-month feeding domain contained nine candidate indicators reflecting different dimensions of early infant nutrition, including breastfeeding modality, complementary food introduction, and dietary quality. To develop an etiologically interpretable model, we conducted a pre-specified correlation analysis to identify the subset of variables that best captured distinct feeding mechanisms while avoiding multicollinearity and redundancy. Several candidate variables showed strong intercorrelations, particularly among indicators of dietary composition (sweet beverage consumption, mixed milk feeding, introduction of solids, and absence of fruit/vegetables; r = 0.48 to 0.54), suggesting these variables measured overlapping aspects of overall dietary quality rather than distinct pathways. In contrast, exclusive breastfeeding and egg/flesh food consumption showed weak-to-negligible correlations with most other measures and with each other (ρ = −0.16), indicating they represented independent feeding constructs. Exclusive breastfeeding <6 months reflects the timing and content of early milk-based nutrition and mechanistically prevents other feeding modes; egg/flesh food consumption represents the introduction and quality of complementary protein-dense foods at six months. This parsimonious two-variable specification reduces overfitting, and improves interpretability.

**Supplementary Material S2. Joint (multivariate) model of weight-for-age and height-for-age z-scores at 12 months**

**Methods**

Weight-for-age (WAZ) and height-for-age (HAZ) are calculated from the same infant’s measurements, so they are not independent outcomes. Analysing them in separate regressions ignores this overlap. We therefore refitted the final six-month model (infant sex, maternal education, exclusive breastfeeding, and egg/flesh-food consumption) as a joint multivariate model, estimating both z-scores together and allowing their errors to correlate. The joint model adds a single test of whether each predictor is related to growth as a whole. Analyses included the 66 infants with complete data.

**Table S2.** Joint multivariate model of weight-for-age (WAZ) and height-for-age (HAZ) z-scores at 12 months, (n = 66).

|  | **WAZ at 12 months** | | **HAZ at 12 months** | | **Joint test** | |
| --- | --- | --- | --- | --- | --- | --- |
| **Predictor** | **B (95% CI)** | **p** | **B (95% CI)** | **p** | **F(2, 60)** | **p** |
| Infant sex (female vs male) | −0.008 (−0.550, 0.533) | 0.975 | 0.326 (−0.245, 0.897) | 0.258 | 1.19 | 0.311 |
| Maternal education any vs none) | 0.528 (−0.083, 1.139) | 0.089 | 0.676 (0.033, 1.320) | 0.040 | 2.27 | 0.112 |
| Exclusive breastfeeding < 6 months | −0.417 (−0.942, 0.108) | 0.118 | −0.613 (−1.166, −0.060) | 0.030 | 2.43 | 0.097 |
| Egg/flesh food consumption (EFF) 6–23 months | 0.550 (−0.106, 1.205) | 0.099 | 0.208 (−0.483, 0.899) | 0.549 | 1.62 | 0.207 |
| *Model R² (single-outcome)* | *0.159* | *0.030* | *0.157* | *0.032* | *-* | *0.041* |

B, unstandardised regression coefficient; CI, confidence interval; WAZ, weight-for-age z-score; HAZ, height-for-age z-score.

Infant sex coded 0 = male (reference), 1 = female; maternal education coded 0 = no formal education (reference), 1 = primary or above; exclusive breastfeeding and egg/flesh-food consumption coded 0 = no (reference), 1 = yes.

The joint test reports Wilks’ lambda for each predictor across both outcomes simultaneously, on 2 and 60 degrees of freedom.

The final row reports the R² and overall model p-value of the corresponding single-outcome regression, shown for reference.

**Discussion**

The two outcomes were strongly correlated at 12 months (Pearson r = 0.697, p < 0.001; n = 66) and remained so after adjustment (residual r = 0.665), confirming that they carry largely overlapping information. Estimating them together left every coefficient unchanged, so the associations reported in the main analysis do not depend on having modelled the two outcomes separately.

Taken as a whole, the joint model was statistically significant (Wilks’ lambda = 0.770; F(8, 120) = 2.09; p = 0.041), indicating that the four predictors together are related to infant growth. Within that model, however, no single predictor reached p < 0.05. Exclusive breastfeeding came closest (p = 0.097), followed by maternal education (p = 0.112). This combination - a significant model containing no individually significant predictor - is a familiar consequence of limited sample size, and indicates that the cohort can demonstrate that these factors matter collectively without being large enough to isolate the contribution of each one.

The individual joint tests are also conservative by construction. A joint test asks a stricter question than a single-outcome test, namely whether a predictor is related to both outcomes at once, and it pays a statistical penalty for testing two outcomes while gaining little additional information from the second, since the two are so highly correlated. The joint model therefore confirms that the estimates remain stable once the correlation between outcomes is taken into account, while the outcome-specific models remain the appropriate basis for judging individual associations.

**Supplementary Material S3. Repeated-measures (random-intercept) mixed-effects sensitivity analysis**

**Methods**

To assess whether the domain-model associations were sensitive to the single-endpoint design, and to account for the correlation among repeated anthropometric measurements taken on the same infant, we fitted random-intercept linear mixed-effects models to all available child-occasion observations across the four visits (0, 3, 6, and 12 months). height-for-age (HAZ) and weight-for-age (WAZ) z-scores were modelled as separate outcomes. Each model included occasion as a categorical fixed effect, a random intercept for each infant (to capture within-infant correlation), and fixed-effect adjustment for infant sex and maternal education (primary or above versus no formal education). Models were estimated by restricted maximum likelihood (REML). The intraclass correlation coefficient (ICC) was obtained from unconditional (intercept-only) models.

Robustness of the maternal-education association was examined in three ways: (i) replacing the infant-level random intercept with nested mother-and-infant random intercepts, which allows the three infants of the single multiple-birth set to be correlated with one another rather than treated as independent; (ii) excluding that triplet set entirely; and (iii) refitting the model as a generalized estimating equation (GEE) with an exchangeable working correlation. The GEE specification was included because, unlike the mixed model, it makes no distributional assumption about the between-infant random effects and uses robust (sandwich) standard errors that remain valid even if the assumed within-infant correlation structure is misspecified. It therefore provides a distribution-free check on the maternal-education association rather than a re-estimate of it.

Analyses were conducted in IBM SPSS Statistics. The HAZ analysis used 264 observations and the WAZ analysis 268 observations, each on 72 infants.

**Table S3.1.** Random-intercept linear mixed-effects models for height-for-age (HAZ) and weight-for-age (WAZ) z-scores across four occasions.

| **Parameter** | **HAZ: B (95% CI)** | **p** | **WAZ: B (95% CI)** | **p** |
| --- | --- | --- | --- | --- |
| *Fixed effects (adjusted)* |  |  |  |  |
| Maternal education (any vs none) | 1.30 (0.60, 1.99) | < 0.001 | 0.25 (−0.21, 0.70) | 0.285 |
| Infant sex (female vs male) | 0.47 (−0.18, 1.11) | 0.153 | 0.18 (−0.23, 0.60) | 0.383 |
| Occasion | F(3, 190) = 9.65 | < 0.001 | F(3, 200) = 48.57 | < 0.001 |
| *Variance structure (unconditional)* |  |  |  |  |
| Between-infant variance | 1.32 |  | 0.11 |  |
| Residual variance | 2.15 |  | 2.29 |  |
| **Intraclass correlation (ICC)** | **0.38** |  | **0.04** |  |
| Observations (infants) | 264 (72) |  | 268 (72) |  |

Estimates for maternal education and infant sex are adjusted fixed-effect coefficients (B) from REML random-intercept models, with occasion entered as a categorical fixed effect and a random intercept for each infant.

Maternal education coded 0 = no formal education (reference), 1 = primary or above; infant sex coded 0 = male (reference), 1 = female. Positive coefficients therefore denote higher z-scores among infants of educated mothers and among female infants. The occasion row reports the Type III omnibus test.

Variance components and the ICC are from unconditional (intercept-only) random-intercept models; ICC = between-infant variance ÷ (between-infant + residual variance). Stable between-infant differences accounted for 38% of the variance in HAZ but only 4% of the variance in WAZ.

**Table S3.2.** Robustness of the maternal-education association with length-for-age (HAZ) across model specifications.

| **Model / specification** | **Obs.** | **B** | **95% CI** | **p** |
| --- | --- | --- | --- | --- |
| Random intercept, infant (primary) | 264 | 1.30 | 0.60, 1.99 | < 0.001 |
| Nested random intercepts, mother + infant | 264 | 1.34 | 0.65, 2.02 | < 0.001 |
| Excluding the triplet set | 252 | 1.47 | 0.80, 2.14 | < 0.001 |
| GEE, exchangeable working correlation | 264 | 1.29 | 0.61, 1.97 | < 0.001 |
| *12-month single endpoint (OLS, reference)* | *66* | *0.70* | *0.06, 1.35* | *0.033* |

All rows model HAZ across the four occasions unless otherwise noted. B is the coefficient for maternal education (primary or above versus no formal education).

Because only one mother in the cohort contributed more than one infant, the mother- and infant-level variance components in the nested specification are only weakly separately identified. That model is therefore reported as a conservative check on the influence of the multiple-birth set, rather than as a formally preferred variance decomposition.

The final row is the 12-month single-endpoint ordinary least-squares estimate, shown for reference. Its smaller coefficient reflects the education gap being widest in early infancy and narrowing by 12 months, so the mixed-model estimate represents an association averaged across infancy rather than a re-estimate of the 12-month effect.

**Interpretation**

The association between maternal education and length-for-age persisted when all four measurement occasions were modelled jointly, and held across every specification examined (B = 1.29 to 1.47; all p < 0.001). It was robust to nested random effects, to exclusion of the triplet set, and to a GEE specification that makes no assumption about the distribution of the random effects. The association is therefore not an artefact of analysing a single endpoint, nor of the within-infant correlation in the data.

The three repeated-measures specifications differ only in how much weight the single triplet set carries, and their estimates order accordingly: 1.30 when the three infants are treated as independent, 1.34 when they are allowed to correlate as siblings, and 1.47 when they are excluded altogether. The gradient is modest and every estimate remains significant, indicating that the multiple-birth set does not drive the finding. Consistent with this, the within-infant correlation estimated empirically by the GEE (0.34) closely matched the compound-symmetry correlation implied by the random-intercept model (0.38), indicating that this assumption was not materially violated and accounting for the near-identical estimates across the two approaches.

Substantial between-infant clustering in HAZ (ICC = 0.38) contrasted with minimal clustering in WAZ (ICC = 0.04), and maternal education was not associated with weight-for-age. This pattern is consistent with the stronger tracking of linear than ponderal growth reported in the main text: height-for-age status was comparatively stable within infants across infancy, whereas weight-for-age fluctuated more markedly across visits. Full growth-velocity modelling was not pursued here, as it would be fragile at the present sample size and with visit-wise missingness; it is designated as a priority for the larger, ongoing two-year CHAMP dataset.

**Supplementary Material S4. Leave-one-cluster-out sensitivity analysis**

**Table S4 The pre-specified final six-month model with and without the triplet set — CHAMP cohort.**

*The final six-month etiologic model (child sex, maternal education, exclusive breastfeeding under six months, and egg/flesh food consumption at six months) was refitted after excluding the one set of triplets, which are statistically non-independent. Coefficients retained their direction and approximate magnitude across both outcomes, indicating that the triplet cluster did not drive the findings.*

| **Predictor** | **Full sample (n = 66)** | | **Excluding triplets (n = 63)** | |
| --- | --- | --- | --- | --- |
|  | **B (95% CI)** | **p** | **B (95% CI)** | **p** |
| **Weight-for-age z-score (WAZ) at 12 months** | **R² = 0.159; model p = 0.030** | | **R² = 0.166; model p = 0.030** | |
| Child sex (female) | −0.008 (−0.550, 0.533) | 0.975 | −0.020 (−0.587, 0.546) | 0.943 |
| Maternal education (any vs none) | 0.528 (−0.083, 1.139) | 0.089 | 0.551 (−0.075, 1.177) | 0.083 |
| Egg/flesh food consumption (EFF) 6–23 months | 0.550 (−0.106, 1.205) | 0.099 | 0.586 (−0.084, 1.255) | 0.085 |
| Exclusive breastfeeding <6 m (EBF) | −0.417 (−0.942, 0.108) | 0.118 | −0.367 (−0.912, 0.179) | 0.184 |
| **Height-for-age z-score (HAZ) at 12 months** | **R² = 0.157; model p = 0.032** | | **R² = 0.173; model p = 0.025** | |
| Child sex (female) | 0.326 (−0.245, 0.897) | 0.258 | 0.410 (−0.172, 0.992) | 0.164 |
| Maternal education (any vs none) | 0.676 (0.033, 1.320) | **0.040** | 0.744 (0.101, 1.387) | **0.024** |
| Egg/flesh food consumption (EFF) 6–23 months | 0.208 (−0.483, 0.899) | 0.549 | 0.267 (−0.422, 0.955) | 0.441 |
| Exclusive breastfeeding <6 m (EBF) | −0.613 (−1.166, −0.060) | **0.030** | −0.531 (−1.091, 0.029) | 0.063 |

B, unstandardised regression coefficient; CI, confidence interval. Each outcome represents a separate multivariable linear regression; all models adjusted for child sex. Egg/flesh food consumption (EFF) is measured at 6 months. The full sample comprises 72 infants from 70 mothers (including one triplet set); the analytic n reflects complete cases on all model variables. Bold p-values are < 0.05. The exclusive breastfeeding-HAZ point estimate was essentially unchanged on exclusion (−0.613 → −0.531) but became borderline (p = 0.030 → 0.063) at the reduced sample size, consistent with the study’s limited power for small effects rather than with confounding by the multiples.

**Supplementary Material S5.**

**Supplementary Table S5. Parsimonious composite model relating the leading correlate from each domain, mutually adjusted, to weight-for-age (WAZ) and height/length-for-age (HAZ) Z-scores at 12 months (n = 63).**

| **Predictor** | **WAZ: B (95% CI)** | **p** | **HAZ: B (95% CI)** | **p** |
| --- | --- | --- | --- | --- |
| Child sex | −0.023 (−0.576, 0.529) | 0.933 | 0.241 (−0.342, 0.825) | 0.411 |
| Maternal education (any vs none) | 0.347 (−0.295, 0.989) | 0.284 | 0.592 (−0.087, 1.270) | 0.086 |
| Exclusive breastfeeding <6 months | −0.436 (−0.983, 0.110) | 0.115 | −0.491 (−1.069, 0.086) | 0.094 |
| Egg/flesh food consumption (EFF) 6–23 months | 0.581 (−0.095, 1.258) | 0.091 | 0.307 (−0.408, 1.022) | 0.393 |
| Diarrhoea (6 months) | 0.272 (−0.272, 0.816) | 0.321 | 0.298 (−0.277, 0.873) | 0.304 |
| *R²* | 0.168 |  | 0.162 |  |
| Overall model (F-test) | p = 0.057 |  | p = 0.067 |  |

*Notes.* Values are unstandardized regression coefficients (B) with 95% confidence intervals from separate multivariable linear regression models for each outcome; all five predictors were entered simultaneously and are therefore mutually adjusted. The analytic sample was 63 infants with complete data on every model variable, giving approximately 12.6 observations per predictor. All variance inflation factors were <1.2, indicating no material collinearity.

^*^ Coding: maternal education, any formal education vs none (reference); exclusive breastfeeding, <6 yes vs no (reference); egg and/or flesh-food consumption measured at 6 months. Diarrhoea, 1 = yes vs 0 = no; child sex coded as in the primary analysis.

^**^ The directions of the maternal-education (positive) and feeding (exclusive breastfeeding negative; egg/flesh-food positive) associations were preserved under mutual adjustment. Neither overall model reached statistical significance, consistent with the reduced power of the fully mutually adjusted specification; estimates are interpreted as hypothesis-generating.
